# Inhibitory effects of Patchouli alcohol on the early lifecycle stages of influenza A virus

**DOI:** 10.3389/fmicb.2022.938868

**Published:** 2023-02-01

**Authors:** Yaohua Fan, Qiong Zhang, Wen Zhang, Yanni Lai, Haishan Long, Huiting Huang, Shaofeng Zhan, Xiaohong Liu, Jielan Lai, Zhongde Zhang, Pan Pan, Ziren Su, Geng Li

**Affiliations:** ^1^Laboratory Animal Center, Guangzhou University of Chinese Medicine, Guangzhou, China; ^2^Shenzhen Hospital of Integrated Traditional Chinese and Western Medicine, Shenzhen, China; ^3^Guangzhou Laboratory, Guangzhou, China; ^4^School of Basic Medical Sciences, Guangzhou University of Chinese Medicine, Guangzhou, China; ^5^Department of Pneumology, The First Affiliated Hospital of Guangzhou University of Chinese Medicine, Guangzhou, China; ^6^Cancer Prevention and Treatment Center, Sun Yat-sen University, Guangzhou, China; ^7^Department of Emergency, The Second Affiliated Hospital of Guangzhou University of Chinese Medicine, Guangzhou, China; ^8^Guangdong Provincial Key Laboratory of Virology, Institute of Medical Microbiology, The First Affiliated Hospital of Jinan University, Guangzhou, China; ^9^Guangdong Provincial Key Laboratory of New Drug Development and Research of Chinese Medicine, Guangzhou University of Chinese Medicine, Mathematical Engineering Academy of Chinese Medicine, Guangzhou, China

**Keywords:** Patchouli alcohol, influenza virus, antiviral, hemagglutination, membrane fusion

## Abstract

**Background:**

The antiviral activity and underlying mechanism of Patchouli alcohol remain unclear.

**Methods:**

This study evaluated the cytotoxicity, optimal methods for drug administration, anti-influenza A activity of Patchouli alcohol. The antiviral mechanism of Patchouli alcohol was also assessed *via* qRT-PCR, western blot, hemagglutination inhibiting (HAI) assay, and hemolysis inhibiting assay.

**Results:**

Patchouli alcohol was shown to have low cytotoxicity and its strongest antiviral effect was associated with premixed administration. Patchouli alcohol inhibited virus replication during the early lifecycle stages of influenza A virus infection and specifically prevented expression of the viral proteins, HA and NP. In both the HAI and hemolysis inhibiting assays, Patchouli alcohol was able to block HA2-mediated membrane fusion under low pH conditions. Patchouli alcohol had lower binding energy with HA2 than HA1.

**Conclusion:**

These findings suggest that Patchouli alcohol could be a promising membrane fusion inhibitor for the treatment of influenza A infection.

## 1. Introduction

Influenza is a highly contagious respiratory disease caused by influenza viruses, enveloped single-stranded negative-stranded RNA viruses of the Orthomyxoviridae family (Bouvier and Palese, 2008). These infections are a substantial threat to human health, resulting in 3–5 million cases of infection and 250,000–500,000 deaths each year (Harper et al., 2004).

The influenza family includes four genera, A, B, C, and D, based on the expression of specific nucleoprotein and matrix protein antigens. Influenza A viruses are further subclassified into subtypes, such as H1N1, H2N2, and H3N2, according to antigenic differences in hemagglutinin (HA) and neuraminidase (NA). Only influenza A viruses can cause pandemics, such as the 2009 influenza H1N1 pandemic, during which nearly a million people were infected worldwide and about 120,000 in China (Salto-Quintana et al., 2019). The highly pathogenic avian influenza (HPAI), including the H5N1 and H7N9 outbreaks of 2013, also caused substantial global morbidity and mortality.

Influenza vaccines and drugs are widely used but their efficacy remains limited by antigenic mutation and drug resistance. The high frequency of antigenic mutations, including antigenic drift and antigenic transfer, as well as the time required to prepare vaccines, has meant that vaccines lag behind the most recent viral mutations (Nishikawa et al., 2012; McKimm-Breschkin, 2013; Wei et al., 2020). Influenza drugs are classified into neuraminidase inhibitors such as Oseltamivir, Zanamivir, and Peramivir, and Matrix 2 ion channel inhibitors, such as amantadine and rimantadine. Long-term use of these drugs is associated with multiple side effects and can promote resistance (Nicholson, 1996; Hayden, 2006; Darwish et al., 2011). Thus, there is an urgency to develop new, low-toxicity influenza treatments.

Traditional Chinese medicine has attracted widespread attention for use in the prevention and treatment of viral infections, especially during the 2003 severe acute respiratory syndrome (SARS) and novel coronavirus disease 2019 (COVID-19) outbreaks. *Pogostemon cablin Benth*, a traditional Chinese herb, has been widely used to treat the “common cold and summer influenza.” This medicine is also able to improve digestive function, removing dampness, analgesic, and anti-inflammatory activities, and inhibiting antiemetic activity (Kiyohara et al., 2012). Patchouli alcohol, one of the primary ingredients of *Pogostemon cablin Benth*, exhibits a potent anti-influenza virus activity and can improve the survival of influenza virus-infected mice (Li et al., 2012).

Studies have shown that Patchouli alcohol can inhibit influenza *in vitro* by downregulating the production of inflammatory factors such as IFN-γ and IL-4 (Wu et al., 2013; Yu et al., 2019). However, the target and mechanism by which Patchouli alcohol exerts its anti-viral effects remain unclear. This study aimed to further evaluate the effect of Patchouli alcohol against influenza viruses. HA2 was used as the target to verify the effect of Patchouli alcohol on the replication of the influenza virus using HA and hemolysis-inhibiting assays.

## 2. Materials and methods

### 2.1. Compound

Patchouli alcohol (purity > 98%) was extracted from *Pogostemon cablin Benth*, kindly provided by Prof. Ziren Su (Guangzhou University of Chinese Medicine). It was dissolved in 100% dimethyl sulfoxide (DMSO) as a stock solution and diluted in various concentrations with serum-free minimum essential medium (MEM). The final concentration of DMSO in the culture medium was 1% for the *in vitro* studies. Oseltamivir phosphate (purity > 98%), known as a neuraminidase inhibitor, was the positive control for anti-influenza viral activity assays and was purchased from Shanghai Yuanye Biotechnology Co., Ltd. It was dissolved in MEM as a stock solution and diluted in various concentrations.

### 2.2. Cells culture and viruses

Madin–Darby Canine kidney (MDCK) cells were donated from the State Key Laboratory of Respiratory Disease (Guangzhou Medical University, Guangzhou, China) and cultured in MEM containing 10% fetal bovine serum (FBS) and 1% penicillin/streptomycin (P/S) at 37°C, 5% v/v CO_2_ in a humidified incubator. Human lung adenocarcinoma 549 (A549) cells and African green monkey kidney (Vero) cells were obtained from Wuhan University (Wuhan, China) and cultured in Dulbecco’s Modified Eagle Medium (DMEM) containing 10% FBS and 1% penicillin/streptomycin (P/S) at 37°C, 5% v/v CO_2_ in a humidified incubator. A/Puerto Rico/8/34 (H1N1), A/FM/1/47 (H1N1), A/WSN/33 (H1N1, S31N, amantadine resistant), A/Chicken/Guangdong/1996 (H9N2), A/HongKong/498/97 (H3N2), and influenza B/Lee/1940 (IVB) were harvested after amplifying in the 37°C embryo allantoic cavity of specific pathogen-free (SPF) embryonated eggs for 48 h. HSV-1 (Shuwen Wu, Wuhan University, Wuhan, China) was propagated in Vero cells. Our laboratory obtained the Patchouli alcohol-tolerant influenza strain through cultivation, and the method is shown in section “2.13. Molecular docking study of the interaction between Patchouli alcohol and HA.” The titer of viruses was measured by plaque assays as described previously (Zhao et al., 2018). The viruses were stored at –80°C in the Laboratory Animal Center of Guangzhou University of Chinese Medicine (Guangzhou, China), and all experiments were performed in Class II biosafety safety cabinets.

### 2.3. Cytotoxicity assays

The cytotoxicity of Patchouli alcohol was assessed by a cell viability assay with a Cell Counting Kit-8 (CCK-8, Dojindo). MDCK, A549, and Vero cells were seeded at 2 × 10^4^ cells/well in 96-well plates and incubated for 18 h. Then cells were incubated with various diluted concentrations of the compound for 48 h, and the cell control group was added to serum-free MEM. Then the medium was replaced with 10% CCK-8 dilution of 100 μl/well and incubated for 30 min; the absorbance was measured with a test 450 nm wavelength by a multimode microplate reader (PerkinElmer).

### 2.4. Methods of drug addition assays

Experiments focused on determining the mode of medication of Patchouli alcohol. Prophylactic administration, premixed administration, simultaneous administration, and therapeutic administration were performed. A/Puerto Rico/8/34 (H1N1) strain of influenza A virus and MDCK cells were used in this part. The medium was removed and washed with PBS; the methods of adding the drugs are shown in Figure 2A. A total of 100 μl/well of 10% CCK-8 dilution was added for 30 min and the absorbance was measured with a test at 450 nm wavelength by a multimode microplate reader.

**FIGURE 1:**
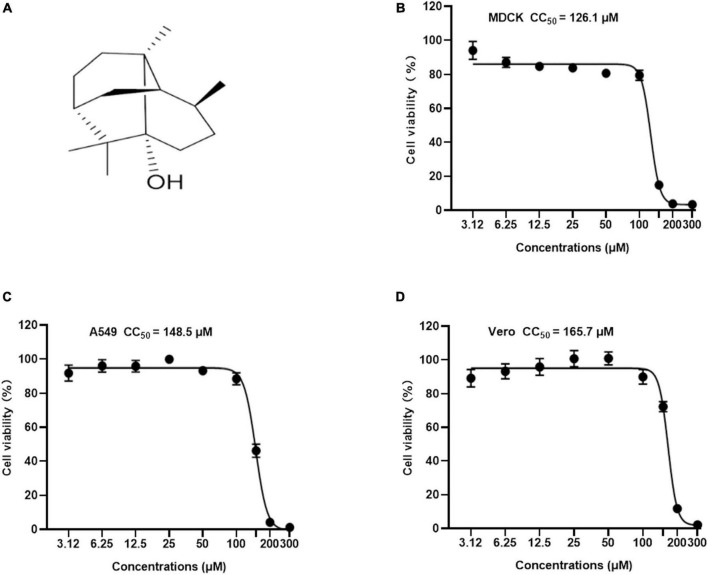
The chemical structure and cytotoxicity of Patchouli alcohol were evaluated by cell viability assay. **(A)** Chemical structure of Patchouli alcohol. **(B)** The CC_50_ of Patchouli alcohol was determined by cell viability assay in MDCK cells. **(C)** The CC_50_ of Patchouli alcohol was determined by cell viability assay in A549 cells. **(D)** The CC_50_ of Patchouli alcohol was determined by cell viability assay in Vero cells. *n* = 3, each concentration conducted in triplicate.

**FIGURE 2:**
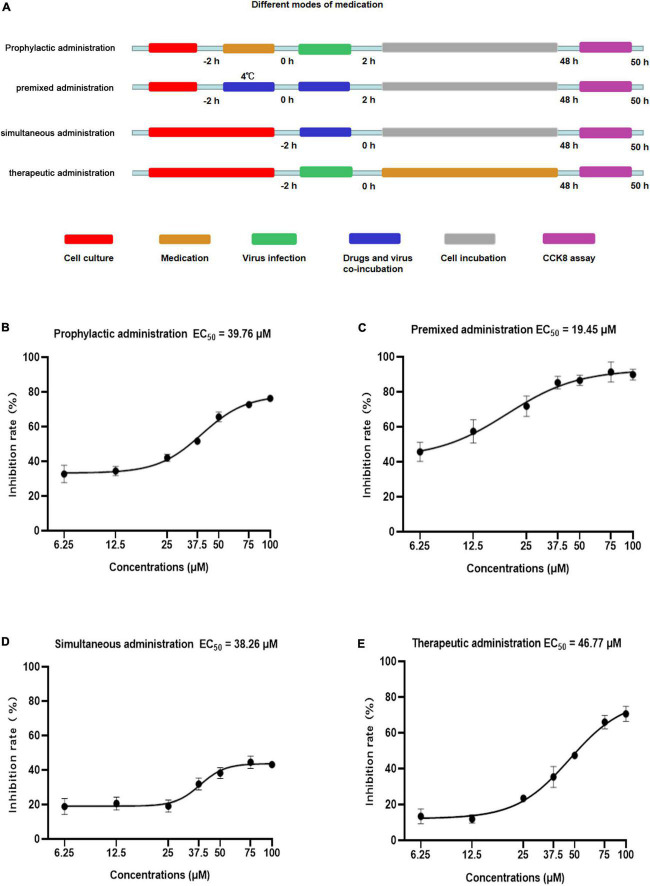
Anti-viral activity of Patchouli alcohol against influenza A virus with different medication modes in MDCK cells. **(A)** Sketch of different medication modes. Prophylactic administration: drugs were added to the cell for incubation before the infection and the virus was added to MDCK cells. Premixed administration: drugs and viruses were mixed and incubated at 4°C for 2 h, then added the infection virus and drug mixture to MDCK cells. Simultaneous administration: drugs and viruses were mixed and immediately added the mixture to the MDCK cell. Therapeutic administration: drugs were added to MDCK cells after infection by the virus. Red: the period of cell culture; orange: the period of drug medication; green: the period of virus infection; blue: drugs and the virus in the medium at the same time; gray: cells were cultured without drugs and virus; purple: the period of detection. **(B)** In prophylactic administration mode, the inhibition rates of Patchouli alcohol on influenza virus A/Puerto Rico/8/34 (H1N1) strain were determined by CCK-8 assay at 48 h post-infection. **(C)** In premixed administration mode, the inhibition rates of Patchouli alcohol on influenza A/Puerto Rico/8/34 (H1N1) strain were determined by CCK-8 assay at 48 h post-infection. **(D)** Simultaneous administration mode and the inhibition rates of Patchouli alcohol on influenza A/Puerto Rico /8/34 (H1N1) strain were determined by CCK-8 assay at 48 h post-infection. **(E)** In therapeutic administration, the inhibition rates of Patchouli alcohol on influenza A/Puerto Rico/8/34 (H1N1) strain were determined by CCK-8 assay at 48 h post-infection. *n* = 3, each concentration was conducted in triplicate.

### 2.5. Anti-influenza viral activity assay

A/Puerto Rico/8/34 (H1N1) strain of influenza A virus was used in the antiviral assays. Vero cells and A549 cells were seeded at 2 × 10^4^ cells/well in 96-well plates. Patchouli alcohol and Oseltamivir phosphate (OS) were diluted with serum-free MEM supplemented with 1 μg of TPCK-trypsin per ml and 1% DMSO, and the virus was diluted with serum-free MEM. Equal volume infection virus medium and various concentrations of drug dilution were incubated at 4°C for 2 h (premixed administration). The blank control group and virus control group were treated with serum-free MEM supplemented with 1 μg of TPCK-trypsin per ml and 1% DMSO. A total of 100 μl/well of 10% CCK-8 dilution was added for 30 min and the absorbance was measured with a test at 450 nm wavelength by a multimode microplate reader. The inhibition rate (%) of Patchouli alcohol was calculated using the following formula:

Inhibition rate (%) = [(mean optical density of test-mean optical density of virus controls) / (mean optical density of cell controls-mean optical density of virus controls)] × 100%

### 2.6. Broad-spectrum anti-viral activity assay

A/FM/1/47 (H1N1), A/WSN/33 (H1N1), A/Chicken/Guangdong/1996 (H9N2), A/HongKong/498/97 (H3N2), influenza B/Lee/1940 (IVB), and HSV-1 strains were used in the antiviral assays. MDCK cells were seeded at 2 × 10^4^ cells/well in 96-well plates. Patchouli alcohol was diluted with serum-free MEM containing 1% DMSO and the virus was diluted with serum-free MEM. Equal volume infection virus medium and various concentrations of drug dilution were incubated at 4°C for 2 h (premixed administration). The blank control group and viral control group were treated with serum-free MEM. The cells were incubated at 37°C supplemented with 1 μg of TPCK-trypsin per ml for 48 h. A total of 100 μl/well of 10% CCK-8 dilution was added for 30 min and the absorbance was measured with a test at 450 nm wavelength by a multimode microplate reader. The inhibition rate of the virus was calculated using the above formula.

### 2.7. Quantitative real-time PCR assay

Madin–Darby Canine kidney cells were seeded at 1 × 10^5^ cells/well in 12-well plates. Patchouli alcohol was serially twofold diluted with serum-free MEM and mixed with virus dilution (A/Puerto Rico/8/34) for 2 h at 4°C containing 1% DMSO and then added to the cell plate for 2 h. Serum-free MEM was replaced with the infection compound and virus mixture at 37°C supplemented with 1 μg of TPCK-trypsin per ml for 48 h. The infected cells were collected and 500 μl of Trizol reagent was added to extract total RNA by the Ultrapure RNA kit (Co Win Biotech, Beijing, China) and cDNA was obtained using the M-MLV Reverse Transcriptase kit (Promega, Madison, WI, United States). The amplification conditions were as follows: 1 cycle at 95°C for 3 min; 39 cycles at 95°C for 10 s; 60°C or 10 s, and 72°C for 20 s; and 1 cycle at 95°C for 10 s. Specific primers were used for the detection and quantification of HA mRNA and NP mRNA (Zhang et al., 2018):

NP forward primer, 5′-GCACCAAACGGTCTTACGAA-3′
NP reverse primer, 5′-TTTGGATCAACCGTCCCTCA-3′
HA forward primer, 5′-TGCTTCCAGTGAGATCATGGTC CTA-3′
HA reverse primer, 5′-GCTGCCGTTACTCCTTTGGTTGT-3′
GAPDH forward primer, 5′-CTTCACCACCATGGAGA AGGCTG-3′
GAPDH reverse primer, 5′-GACCACAGTCCATGCCAT CACTG-3′

The relative expression was measured using CFX connect qRT-PCR instrument.

### 2.8. Western blot assay

Western blot was used for the exploration of the effect of Patchouli alcohol on inhibiting viral protein expression. MDCK cells were seeded at 2 × 10^5^ cells/well in 6-well plates. Patchouli alcohol was serially twofold diluted with serum-free MEM and mixed with virus dilution (A/Puerto Rico/8/34) containing 1% DMSO for 2 h at 4°C and then added to the cell plate for 2 h. Serum-free MEM was replaced with the infection compound and virus dilution at 37°C supplemented with 1 μg of TPCK-trypsin per ml for 48 h. The infected cells were collected and lysed with lysis buffer; the viral protein was collected by centrifugation at 10,000 × *g* for 10 min at 4°C and boiled at 100°C for 10 min. The denatured protein was separated by 10% sodium dodecyl sulfate-polyacrylamide gel electrophoresis, transferred onto a polyvinylidene difluoride (PVDF) membrane, blocked with 5% skim milk, and sequentially incubated with anti-NP and GAPDH primary antibodies (GeneTex, San Antonio, USA), followed by incubation with a secondary antibody. The targeted bands were analyzed using Fluor Chem R multifunctional imaging machine (protein simple, USA).

### 2.9. Time-of-addition approach

Experiments focusing on the effects of Patchouli alcohol on different stages of viral replication were carried out. Patchouli alcohol was serially twofold diluted with serum-free MEM containing 1% DMSO. A total of 2 × 10^5^ MDCK cells per well were seeded in 96-well plates. The confluent monolayer cells infected with the influenza virus were medicated at different time intervals. A total of 50 μl virus or 100 μl drug and virus mixture (A/Puerto Rico/8/34, 100 pfu) for 2 h and 100 μl compound dilutions (100, 50, 25, and 12.5 μM) were incubated at –2 to –1 h,–1 to 0 h, 0–2 h, 2–4 h, 4–6 h, and 6–48 h time intervals, and the absorbance was measured according to the manufacturer’s instructions of a Cell Counting Kit-8 (Dojindo).

### 2.10. Culture and sequence comparison of Patchouli alcohol resistant strains

Patchouli-alcohol-resistant strains were obtained by serial passaging of Influenza A virus (A/Puerto Rico/8/34) in MDCK cells with gradually increasing concentrations of Patchouli alcohol. A total of 2 × 10^5^ MDCK Cells were initially infected with Influenza A virus (A/Puerto Rico/8/34) at MOI 0.3 (passage 1: P1) for 1 h followed by treatment with 5 μM Patchouli alcohol for 48 h. For subsequent passaging, 1 ml of the virus inoculum from the previous passage was used for infection. Patchouli alcohol treatment was gradually increased from 5 to 15 μM for resistance selection, with an increment of 5 μM after every 2 passages. Influenza A virus passaged in 0.1% DMSO (v/v) was included as a mocked-treated control. Reduced Patchouli alcohol susceptibility (defined as a rebounce in virus titer similar to mocked-treated control) was determined from the supernatants collected at 48 h post-infection by plaque assay. Then, we obtained the Patchouli-alcohol-tolerant influenza strain. The RNA from the supernatant of the drug-resistant virus was extracted for gene cloning. The specific primers used for HA were as follows:

Plenti-HA forward primer, 5′-TATCTAGAAAAATGAAGGCA AACCTACTGC-3′
Plenti-HA reverse primer, 5′-CGGGATCCCTCATATCTC TGAAATTCTAA -3′

Then, the PCR solution was sent to Beijing Qingke Biotechnology Co., Ltd. for bidirectional DNA sequencing. Low-mass sequences and primer regions were removed using GeneStudio v.2.2.0.0, and images of the peaks obtained by sequencing were spliced and proofread. Then, an online search in the NCBI database^1^ was performed to obtain the sequences that were the most similar to those of the samples.

### 2.11. Hemagglutination inhibiting assay (HAI)

The hemagglutination inhibiting (HAI) assay was carried out to assess whether Patchouli alcohol inhibits hemagglutination activity. Hemagglutination titers of the A/Puerto Rico/8/34 strains were determined in a U-shape 96-well plate by standard assays, and the unit of hemagglutination agglutination (HAU) was obtained. Patchouli alcohol was serially twofold diluted with phosphate buffer solution (PBS, pH 7.4) containing 1% DMSO (100∼3.125 μM). A total of 25 μl of different concentrations of the compound was mixed with an equal volume of 4 HAU units of influenza virus in PBS and incubated for 2 h at 4°C and then 50 μl of 1% freshly chicken red blood cells preparation was added to each well. After 30 min incubation, the virus titer was measured according to the HAI assay. Control groups for the assay included the virus and PBS mixture containing 1% DMSO and 1% PBS alone (Cetina-Montejo et al., 2019).

### 2.12. Hemolysis inhibiting assay

A hemolysis-inhibiting assay was performed to test and verify the interaction between the HA2 subunit and compound. Patchouli alcohol was serially twofold diluted with PBS (pH 7.4) containing 1% DMSO. Then 100 μl of various concentrations of the compound/PBS and an equal volume (100 μl) of undiluted influenza virus stock sample (A/Puerto Rico/8/34) were mixed and added to each 2-ml well of a V-shaped 96-well plate and incubated for 2 h in 4°C; 200 μl of 1% fresh chicken red blood cells were added to each well and incubated for 10 min at room temperature. Subsequently, centrifugation was done at 1,000 rpm/min for 15 min and the supernatant was thrown. Then, 450 μl acetic acid–sodium acetate (pH5.0) was added and completely mixed to resuspend the chicken red blood cells, and then the plate was incubated for 30 min at room temperature. After centrifugation at 1,000 rpm/min for 15 min, 300 μl supernatant was removed to a new 96-well plate and the absorbance was detected with a test of 540-nm wavelength by a multimode microplate reader. The hemolytic inhibition rate of Patchouli alcohol was calculated from the formula:

$$Inhibitionrate\left(\%\right)=\left[1-\frac{\left(O\,D\,540_{D\,r\,u\,g}-O\,D\,540_{P\,B\,S}\right)}{\left(O\,D\,540_{V\,i\,r\,u\,s}-O\,D\,540_{P\,B\,S}\right)}\right]\times 100\%$$

### 2.13. Molecular docking study of the interaction between Patchouli alcohol and HA

The protein database^2^ was used to obtain the structure of HA (1RU7). Water molecules were removed with PyMOL 2.5. The structure of the Patchouli alcohol was retrieved from the PubChem database.^3^ ChemDraw software was used to create all derived connections in the mol file. The structures were first called up in SDF format and then translated into PDB format using PyMOL. The molecular docking simulations were performed using Auto Dock (The Scripps Research Institute, La Jolla, CA, USA). The docked posture with the best negative score was selected as the best for the appropriate chemical and protein after the docking simulation. PyMOL was used to display the best-docked posture to study unbound interactions.

### 2.14. Statistical analysis

The experimental results were performed in at least 3 replicates. For each experiment, analyses were performed using a one-way analysis of variance (ANOVA), and the mean and standard deviation (SD) were calculated with SPSS 20.0. The *p*-value of < 0.05 means the difference is statistically significant. The 50% cytotoxic concentrations (CC_50_) and 50% effective concentrations (EC_50_) were calculated by non-linear regression using GraphPad Prism 8.0.

## 3. Results

### 3.1. Patchouli alcohol has low cytotoxicity

Patchouli alcohol is a natural tricyclic sesquiterpene isolated from *Pogostemon cablin Benth* (Figure 1A). To identify a safe drug that could potentially work as an antiviral, the cytotoxicity of Patchouli alcohol from 3.12 to 300 μM was assessed in MDCK, A549, and Vero cells. The 50% cytotoxicity concentration (CC_50_) values of Patchouli alcohol were 126.1, 148.5, and 165.7 μM in MDCK, A549, and Vero cells, respectively (Figures 1B–D).

### 3.2. Activity of different Patchouli alcohol administration types against influenza A virus

The activity of four types of Patchouli alcohol administration, prophylactic, premixed, simultaneous, and therapeutic, against influenza virus were compared (Figure 2A). The 50% effective concentration (EC_50_) values were 39.76, 19.45, 38.26, and 46.77 μM, respectively, at doses ranging from 6.25 to 100 μM (Figures 2B–E). The selectivity index (SI) was determined based on the CC_50_ and EC_50_ values (Table 1). Patchouli alcohol displayed higher anti-influenza virus activity using premixed administration than other administration types, effectively demonstrating that Patchouli alcohol can protect cells from virus-induced cell death. Thus, premixed administration was used to further explore the antiviral effect of Patchouli alcohol on different cell types infected with influenza viruses.

**TABLE 1:** Antiviral activity of Patchouli alcohol in MDCK cells.

Drug	Administration modes	CC_50_ = 126.1 (μM)	
		EC_50_ (μM)	SI
Patchouli alcohol	Prophylactic administration	39.76	3.17
	Premixed administration	19.45	6.48
	Simultaneous administration	38.26	3.30
	Therapeutic administration	46.77	2.70

CC_50_, the 50% cytotoxic concentration; EC_50_, the 50% effective inhibitory concentrations; SI, selective index, CC_50_/EC_50_.

### 3.3. The antiviral effects of Patchouli alcohol on A/Puerto Rico/8/34 (H1N1) and other virus strains

**FIGURE 3:**
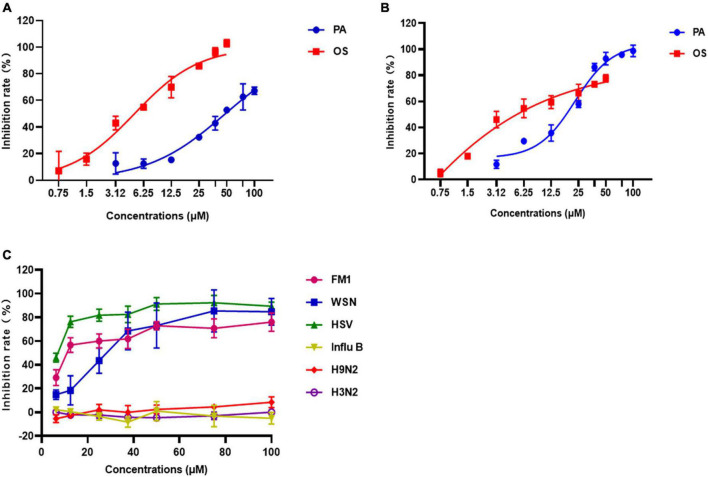
The antiviral effects of Patchouli alcohol on A/Puerto Rico/8/34 (H1N1) strain and other strains of influenza viruses. **(A)** Inhibitory effects of various concentrations of Patchouli alcohol against A/Puerto Rico/8/34 (H1N1) strain influenza virus in Vero cells. Oseltamivir as a positive control. **(B)** Inhibitory effects of various concentrations of Patchouli alcohol against A/Puerto Rico/8/34 strain influenza virus in A549 cells. Oseltamivir as a positive control. **(C)** Inhibitory effects of various concentrations of Patchouli alcohol against A/FM/1/47 (H1N1), A/WSN/33 (H1N1, S31N, amantadine resistant), A/Chicken/Guangdong/1996 (H9N2), A/HongKong/498/97 (H3N2), and influenza B/Lee/1940 (IVB) in MDCK cells. *n* = 3, each concentration conducted in triplicate.

The EC_50_ values of Patchouli alcohol cells were 48.30 μM and 22.99 μM in A/Puerto Rico/8/34 strain (H1N1)-infected Vero and A549 cells, respectively, indicating that Patchouli alcohol had strong anti-influenza activity (Figures 3A, B). Meanwhile, the EC_50_ values of Oseltamivir were 4.981 and 0.07 μM in infected Vero and A549 cells, respectively (Figures 3A, B).

To determine whether Patchouli alcohol has antiviral effects on other viruses, the inhibition rate of Patchouli alcohol in MDCK cells infected with A/FM/1/47(H1N1), A/WSN/33(H1N1), A/Chicken/Guangdong/1996 (H9N2), A/Hong Kong/498/97(H3N2), influenza B/Lee/1940, or HSV-1 was assessed. The inhibition rates were 91.36 ± 5.77%, 75.93 ± 7.76%, 85.32 ± 11.26%, and 92.25 ± 6.25% in cells infected with A/PR/8/34(H1N1), A/FM/1/47(H1N1), A/WSN/33(H1N1) and HSV-1 virus, respectively (Figures 2C, 3C and Table 2). Patchouli alcohol had a stronger antiviral activity against the HSV-1 virus than the A/FM/1/47(H1N1) or the A/WSN/33(H1N1) virus. In contrast, Patchouli alcohol had no obvious inhibitory effects on the A/Chicken/Guangdong/1996(H9N2), A/Hong Kong/498/97(H3N2), or influenza B/Lee/1940 virus.

**TABLE 2:** Patchouli alcohol inhibition of several influenza virus strains and HSV-1.

Drug	Virus strains	CC_50_ = 126.1 (μM)	
		EC_50_ (μM)	SI
Patchouli alcohol	A/FM/1/47(H1N1)	12.13	10.13
	A/WSN/33(H1N1)	27.80	4.54
	HSV-1	12.07	10.44
	InfluBinfluenza B/Lee/1940	>500	<1
	A/Chicken/Guangdong/1996(H9N2)	>500	<1
	H3N2A/Hong Kong/498/97(H3N2)	>500	<1

CC_50_, the 50% cytotoxic concentration; EC_50_, the 50% effective inhibitory concentrations; SI, selective index, CC_50_/EC_50_.

### 3.4. Patchouli alcohol attenuates influenza A virus mRNA transcription and protein expression in MDCK cells

**FIGURE 4:**
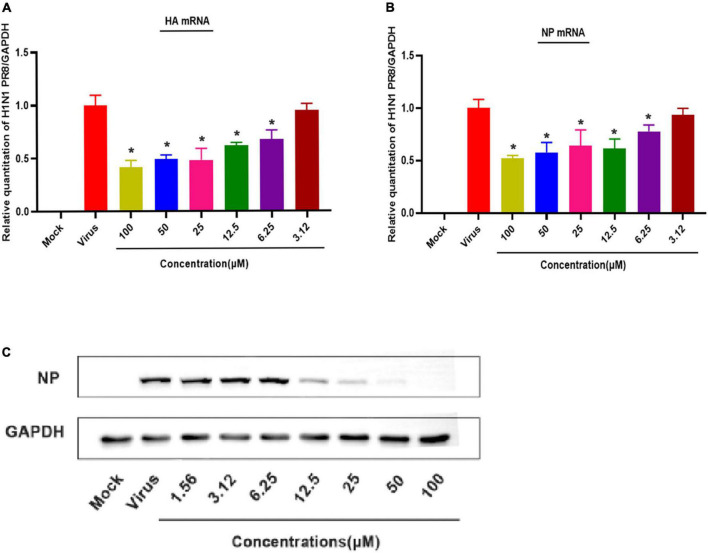
Patchouli alcohol attenuates mRNA transcription and protein expression of the influenza A virus in MDCK Cells. **(A)** The inhibitory effect of Patchouli alcohol on the transcription of influenza A/Puerto Rico/8/34 (H1N1) strain HA mRNA was detected by qRT-PCR at 48 h post-infection. *n* = 3, each concentration conducted in triplicate. *P < 0.05, compared to the viral control group. **(B)** The inhibitory effect of Patchouli alcohol on the transcription of influenza A/Puerto Rico/8/34 (H1N1) strain NP mRNA was detected by qRT-PCR at 48 h post-infection. *n* = 3, each concentration conducted in triplicate. *P < 0.05, compared to the viral control group. **(C)** The inhibitory effect of Patchouli alcohol on the expression of influenza A/Puerto Rico/8/34 (H1N1) strain NP protein was detected by western blot at 48 h post-infection. *n* = 3, each concentration conducted in triplicate.

QRT-PCR was used to assess the effect of different Patchouli alcohol concentrations on viral mRNA expression in MDCK cells infected with the influenza A/Puerto Rico/8/34 (H1N1) strain. HA and NP mRNA expressions at 48 h post-infection were lower in the Patchouli-alcohol-treated group than the control group (*P* < 0.05) (Figures 4A, B). Similarly, viral NP protein expression was completely inhibited by Patchouli alcohol concentrations of 12.5–100 μM (Figure 4C). These results suggested that Patchouli alcohol can make fewer cells infected with the influenza virus.

### 3.5. Inhibitory effects of Patchouli alcohol on the early lifecycle stages of influenza virus

**FIGURE 5:**
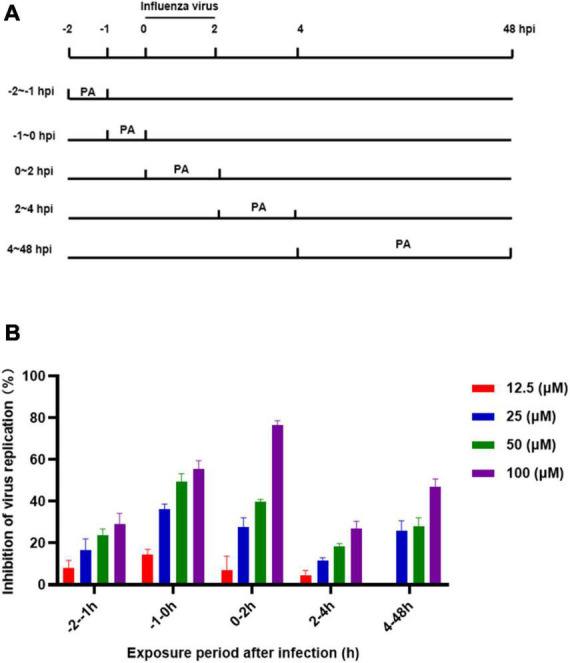
Patchouli alcohol attenuates influenza virus A/Puerto Rico/8/34 (H1N1) strain replication at the early stage of its lifecycle. **(A)** The sketch of the time of medication addition. **(B)** Cells were inoculated with 0.1 mL/well (100 pfu) influenza virus in a time-of-addition assay. 12.5∼100 μM of Patchouli alcohol was added at the designated time interval and removed after each incubation period. The cells were incubated with the fresh medium until 48 h post infection. The virus replication was determined through a CCK-8 assay. *n* = 3, each concentration conducted in triplicate.

The complete life cycle of the influenza virus, including surface binding, virus entry, genome uncoating, transcription/replication, and virus release, takes approximately 8 h. At least 6 h are required to detect progeny after initial incubation of the influenza virus. To explore the antiviral mechanism of Patchouli alcohol, a time-of-addition assay was used to estimate the inhibition of virus replication by treating the virus with Patchouli alcohol at different lifecycle stages. The inhibition rate of virus replication was assessed in five time intervals: –2 to –1 h, –1–0 h, 0–2 h, 2–4 h, and 4–48 h (Figure 5A). Patchouli alcohol (100 μM) had the strongest inhibitory effect on influenza at the -1 to 0 h and 0–2 h time periods, with inhibition rates of 55.57 and 76.7%, respectively (Figure 5B). The inhibitory effect of Patchouli alcohol was the lowest (30%) during the 2–4 h time period (Figure 5B).

### 3.6. Inhibitory effect of Patchouli alcohol on HA-mediated hemolysis of influenza virus

**FIGURE 6:**
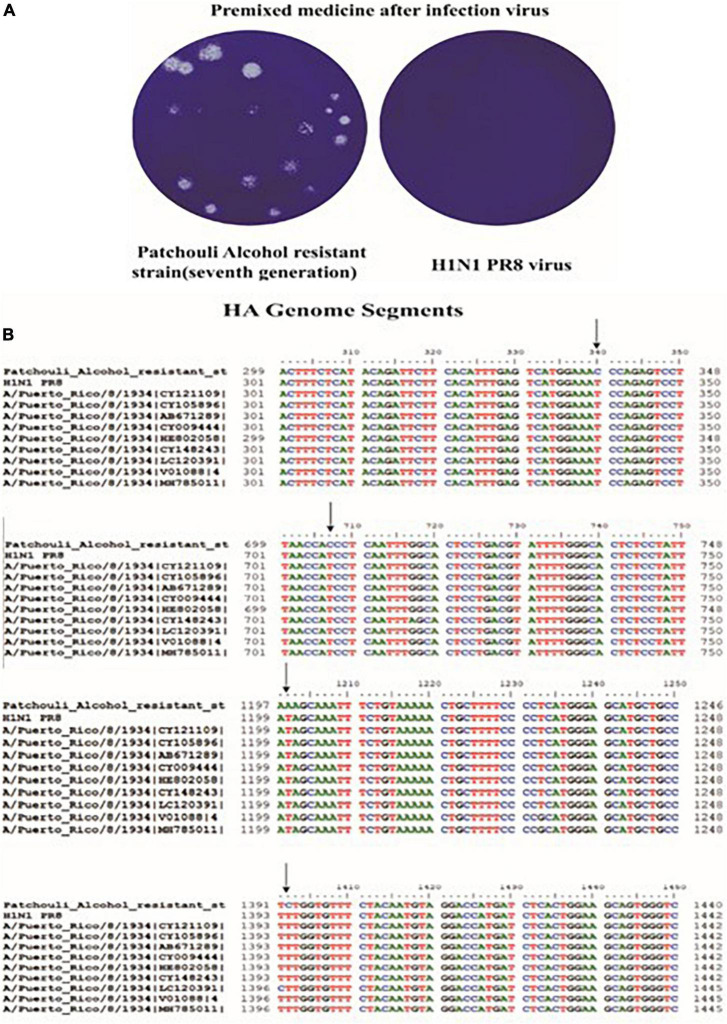
Culture and sequence comparison of Patchouli-alcohol-resistant strains. **(A)** Patchouli-alcohol-resistant strains detected by plaque reduction assay. **(B)** Sequence comparison of Patchouli-alcohol-resistant strains.

Patchouli alcohol was able to inhibit influenza virus replication at 100 μM. We obtained a Patchouli-alcohol-tolerant influenza strain in P7 supernatant, which can significantly resist the antiviral effect of Patchouli alcohol (Figure 6A). In addition, a 4-base mutation was identified in the HA gene sequence of a Patchouli-alcohol-tolerant influenza strain (Figure 6B, Table 3, and Supplementary material). These results suggested that Patchouli alcohol directly inactivates the influenza virus by interacting with HA.

**TABLE 3:** Base and amino acid mutation sites of the HA gene in a Patchouli alcohol-resistant influenza strain.

Base	Nucleotide	Amino acid
340	T→C	Ser→Pro
707	T→C	Ile→Thr
1,202	T→A	His→Gln
1,402	T→C	Phe→Ser

**FIGURE 7:**
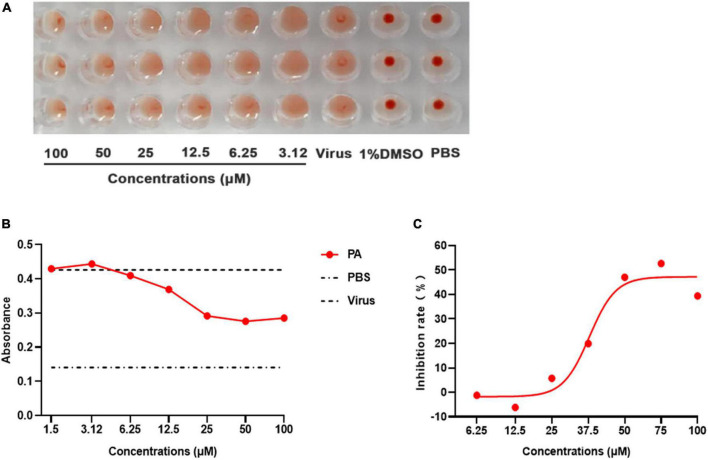
Patchouli alcohol inhibits influenza A virus HA-mediated hemolysis. **(A)** The hemagglutination inhibiting assay. 25 μL influenza virus A/Puerto Rico/8/34 (H1N1) strain was incubated with equal volume serially twofold Patchouli alcohol dilution at 4°C for 2 h, then 50 μL of a 1% suspension of chicken red blood cells were added and incubated for 1 h at room temperature. The blank control was a PBS solution, *n* = 3, each concentration was conducted in triplicate. **(B)** Hemolysis inhibition assay of Patchouli alcohol against influenza virus. 100 μL influenza A/Puerto Rico/8/34 (H1N1) strain virus was mixed in a 96-well-plate with an equal volume of serially twofold Patchouli alcohol dilution and incubated, 2% chicken red blood cell suspension was added after incubation and centrifuged. Then, sodium acetate at pH 5.0 was added and incubated to bring about hemolysis. The OD values were detected at 540 nm wavelength by a multimode microplate reader, *n* = 3, and each concentration was conducted in triplicate. **(C)** The inhibition rate of hemolysis of influenza A/Puerto Rico/8/34 (H1N1) strain virus at pH 5.0 by Patchouli alcohol, *n* = 3, each concentration conducted in triplicate.

HA is critical for influenza replication, endocytosis, and fusion. Thus, an HA inhibition assay was used to assess the impact of Patchouli alcohol on the interaction between HA and cellular receptors, a process required for viral attachment to the cell surface. From 3.125 to 100 μM concentrations, Patchouli alcohol was unable to significantly inhibit red blood cell agglutination, indicating that this drug could not effectively inhibit viral attachment (Figure 7A).

A hemolysis assay was designed using the A/Puerto Rico/8/34 (H1N1) strain to determine the effect of Patchouli alcohol on viral fusion. The virus–cell suspension was briefly acidified to different pHs (4.85–6.0) to initiate HA conformational changes capable of lysing chicken red blood cells (cRBCs). The hemolytic inhibition assay showed that Patchouli alcohol had a significant effect on hemolysis caused by the influenza A virus at pH 5.0, an inhibition rate of about 50%, and in a concentration-dependent manner (Figures 7B, C). These results suggested that Patchouli alcohol interacts with the HA2 polypeptide of the influenza HA protein.

**FIGURE 8:**
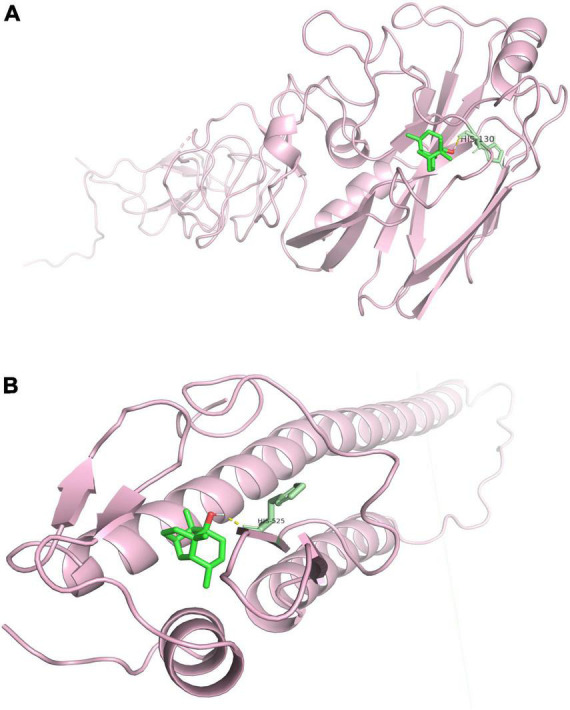
Molecular interactions between Patchouli alcohol and HA. **(A)** Molecular interactions between Patchouli alcohol and HA1. **(B)** Molecular interactions between Patchouli alcohol and HA2.

The binding energy between Patchouli alcohol and HA2 was lower than the binding energy between Patchouli alcohol and HA1 (-7.07 kcal/mol vs. -4.87 kcal/mol, respectively) (Table 4). Patchouli alcohol and HA1 were shown to interact through HIS-130 (Figure 8A), and Patchouli alcohol was also found to form one hydrogen bond with HIS-525, the primary inhibiting site on HA2 (Figure 8B).

**TABLE 4:** *In silico* Patchouli alcohol and HA docking studies.

Compounds	Protein	Binding energy (kcal/mol)	Interactions with amino acid
Patchouli alcohol	HA1	–4.87	HIS-130
	HA2	–7.07	HIS-525

## 4. Discussion

Influenza is associated with high morbidity and mortality rates and remains a serious threat to human health. Infection can lead to a variety of complications, including heart failure, viral pneumonia, viral lung injury, and acute respiratory distress syndrome (ARDS) (Reed et al., 2014; Klomp et al., 2021) that can result in serious sequelae and even death (Peteranderl et al., 2016; Chow et al., 2019). As a result, influenza has attracted the focus of researchers throughout the world. Patchouli alcohol, a volatile oil ingredient extracted from the Traditional Chinese Medicine herb, *Pogostemon cablin Benth*, is shown to have multiple antiviral, immunomodulatory, anti-inflammatory, antioxidative, antitumor, antimicrobial, insecticidal, antiatherogenic, antiemetic, whitening, and sedative effects (Hu G. et al., 2017). Our team has proved in previous articles that Patchouli alcohol has an antiviral effect *in vivo* (Li et al., 2012). Also, it is reported that the inhibition effects of Patchouli alcohol against influenza A virus is through targeting cellular PI3K/Akt and ERK/MAPK signaling pathways, and the RLH signal pathway (Wu et al., 2013; Yu et al., 2019). However, there are few studies on the efficacy and mechanism of Patchouli alcohol directly inhibiting the influenza A virus.

The current study found that Patchouli alcohol had limited cytotoxicity in MDCK, A549, and Vero cells (Figures 1B–D). Premixed administration of Patchouli alcohol was shown to significantly inhibit viral activity in Vero and A549 cells, both of which are susceptible to the influenza virus (Chua et al., 2019). The EC_50_ values were 43.09 and 22.99 μM in Vero and A549 cells, respectively (Figures 3A, B). MDCK, Vero, and A549 cells are derived from dogs, monkeys, and humans, respectively, potentially explaining why Patchouli alcohol activity differs between them. Broad-spectrum anti-viral activity assays were used to determine whether Patchouli alcohol could also inhibit other viruses. Although we proved for the first time that Patchouli alcohol has no obvious effect on H9N2 and H3N2 viruses, it was shown to have strong anti-viral activity against both H1N1 and HSV-1, suggesting that Patchouli alcohol has an antiviral function against a variety of viruses (Figure 3C).

To further explore the mechanism by which Patchouli alcohol inhibits influenza A virus activity, HA and NP expression were evaluated. HA aids influenza virus replication, facilitates viral binding to host cell receptors, and induces viral fusion, while NP is a structural protein with no intrinsic enzymatic activity that plays a central role in viral replication. NP is also the most abundant viral protein in infected cells (Hu Y. et al., 2017; Wu et al., 2017). Although a previous study found that Patchouli alcohol can interfere with the NA functions against the influenza A (H2N2) virus (Wu et al., 2011), our study revealed that Patchouli alcohol could make fewer cells infected with the influenza virus by repressing influenza A virus HA and NP mRNA and inhibiting NP protein expression (Figures 4A–C). These results confirmed that Patchouli alcohol has a direct inhibitory effect on the influenza A virus.

Method-of-addition and time-of-addition studies were conducted to identify which steps of the influenza lifecycle are inhibited by Patchouli alcohol. While prophylactic administration of a drug assesses its effect on cells, premixed administration assesses its effect on the direct killing of the virus at 4°C, simultaneous administration assesses its effect on the virus at 37°C, and therapeutic administration assesses its effect on virus replication (Yu et al., 2017). Results from the current study showed that premixed administration of Patchouli alcohol had a greater inhibition rate on influenza A than prophylactic, simultaneous, or therapeutic administration (Figure 2). These findings suggested that Patchouli alcohol can directly kill influenza viruses and that its inhibitory effect may be directed toward the early stages of viral replication. The replication lifecycle of the influenza virus is roughly divided into adsorption, endocytosis, membrane fusion, replication, assembly, budding, and release, and at least 6 h are required to detect progeny after influenza virus inoculation (Hutchinson, 2018). Time course analysis showed that Patchouli alcohol was able to block influenza A virus replication at the 0–2 h phase of the virus lifecycle, when adsorption, endocytosis, and membrane fusion occur (Figure 5; Makau et al., 2017). These findings verified that Patchouli alcohol is primarily involved in inhibiting the early stages of influenza virus replication.

Influenza virus is a type I enveloped virus whose entry into the target cell is mediated by HA on the viral envelope (Yang et al., 2013). HA, which consists of HA1 and HA2, is a major membrane glycoprotein that plays a significant role in promoting endosomal fusion and allowing for the release of viral genomes during the early stages of replication. HA1 binds with sialic acid on the host cell and mediates viral pinocytosis into the cytoplasm while HA2 mediates viral membrane fusion with the intracellular membrane (Mair et al., 2014; Zhou et al., 2014; Benton et al., 2020). HA2 mainly consists of α. It is composed of a spiral structure. The N terminal contains 23 hydrophobic peptides required for membrane fusion, with two repeated allosteric peptides in the middle. About 28 amino acids are anchored on the membrane at the C terminal, followed by the tail of the intracellular region of 10 residues (Stevens et al., 2004). When HA matures, HA0 is hydrolyzed and cut into HA1 and HA2 by a protease to make the virus infectious and determine its ability to spread in infected host tissues. In highly pathogenic avian influenza virus H5N1 and H7N9 subtypes, HA protein contains several consecutive base amino acid residues at its cutting site, which are very easy to be cut by subtilis proteases, such as Flynn protease. Since these proteases are ubiquitous in many cells, a highly pathogenic avian influenza virus with HA with a multibase cutting site can be produced in most host organs in the form of infection, determining the high pathogenicity of the virus. This cleavage enables HA molecules to undergo great conformational rearrangement in a low PH environment, leading to the exposure of the hydrophobic N-terminal of the HA2 peptide, which can be inserted into the host membrane and anchored. Thus, HA is the main antigen component of the virus. Current findings illustrated that Patchouli alcohol affects four nucleotide sites after binding with the influenza A virus, thus reducing viral activity (Figure 6). The influenza virus HA receptor is present on the surface of chicken red blood cells. Both HA-inhibiting and hemolysis-inhibiting assays demonstrated that Patchouli alcohol may exert its antiviral activity by interacting with the HA2 polypeptide of the influenza HA protein (Figures 7, 8).

The current study has some limitations. Patchouli alcohol showed medium antiviral activity, suggesting that it may function as a lead compound in the development of novel anti-viral drugs. In addition, we used molecular docking to explore the specific target of Patchouli alcohol and HA protein, but further experimental verification is required.

## 5. Conclusion

Patchouli alcohol, a natural compound from *Pogostemon cablin Benth*, was shown to inhibit the early lifecycle stages of the influenza A virus by specifically interacting with HA2 and blocking the fusion of the viral membrane with the intracellular membrane. These findings suggest that HA could serve as a potential antiviral agent.

## Data availability statement

The original contributions presented in this study are included in the article/Supplementary material, further inquiries can be directed to the corresponding authors.

## Author contributions

GL and ZS: conceptualization. JL: methodology. YF: software, formal analysis, and writing—review and editing. QZ, WZ, and HL: validation. PP: investigation. GL: resources and project administration. QZ: data curation and writing—original draft preparation. ZZ: revise draft. YL: visualization. HH and SZ: supervision. GL and XL: funding acquisition. All authors read and agreed to the published version of the manuscript.

## Footnotes

1
https://blast.ncbi.nlm.nih.gov/Blast.cgi

2
https://www.rcsb.org/

3
https://pubchem.ncbi.nlm.nih.gov/compound/10955174
