# Supplementary material for: Inhibitory effects of Patchouli alcohol on the early lifecycle stages of influenza A virus
Source: Front Microbiol. 2023 Feb 1;13:938868. doi: 10.3389/fmicb.2022.938868 (PMC9928722; doi:10.3389/fmicb.2022.938868)
Supplement: Supplementary file 1 [file Table_1.DOCX]

Supplementary Material

**Inhibitory effects of Patchouli alcohol on the early lifecycle stages of influenza A virus**

**Yaohua Fan1†, Qiong Zhang2†，Wen Zhang1†，Yanni Lai3，Haishan Long1，Huiting Huang4，Shaofeng Zhan4，Xiaohong Liu4，Jielan Lai5, Pan Pan6*，Ziren Su7*，Geng Li1***

*To whom correspondence should be addressed.

***Correspondence:**

Geng Li

lg@gzucm.edu.cn;

Ziren Su

suziren@gzucm.edu.cn;

Pan Pan

294593945@qq.com

Gene sequencet

> HA Gene Sequence of Patchouli alcohol resistant Strain 7

CATGCTATTCTGCACTGCAAGATCCATTAGAACACATCCAGAAACTGATTGCCCCCAGGGAGACCAAAAGCACCAGTGAACTGGCGACAGTTGAGTAGATCGCCAGAATCTGATAGATCCCCATTGATTCCAATTTCACTCCATCTACCTTTTCCCTGTTCAACTTTGACTCTTCTGAATATTTGGGATAATCATAAGTCCCATTTCTTACACTTTCCATGCATTCATTGTCACACTTGTGGTAGAACTCAAAACATCCATTTCCGATTTCTTTGGCATTATTCTTTAATTGGCTTTTTACTTTCTCATACAGATTCTTCACATTTGAGTCATGGAAACCCAGAGTCCTTTCATTTTCCAGTAGAACTAACAATTCTGCATTATATGTCCAAATGTCCAGAAATCCATCATCAACTTTTTTATTTAAATTTTCCATCCTTTTTTCTAATTTGTTGAATTCTTTACCCACAGCTGTGAATTGAATGTTCATTTTCTCGATAACAGTGTTCACCTTGTTTGTAATCCCGTTAATGGCATTTTGTGTGCTTTTTTGATCCGCTGCATAGCCTGATCCCTGTTCATTCTGATGATGATAACCATACCATCCATCTATCATTCCAGTCCATCCCCCTTCAATAAAACCGGCAATGGCTCCAAATAGACCTCTGGATTGAATGGACGGAATGTTCCTTAGTCCTGTAACCACCCTCAATTTGGCACTCCTGACGTATTTTGGGCACTCTCCTATTGTGACTGGGTGTATATTCTGGTAAGGGAGACTGCTGTTTATAGCTCCCAGGGGTGTTTGACACTTCGTGTTACACTCATGCATTGATGCGTTTGAGGTGATGATGCCGGACCCAAAGCCTCTACTCAGTGCGAAAGCATACATTGGTGCTATTAGATTTCCATTTGCCTCAAATATTATTGTGTCTCCGGGTTTTAGCAAGGTCCAGTAATAGTTCATCCTCCCAGCTTGATCTCTTACTTTGGGTCTTTCTGCTATTTCCGGGGTAAATCTCCTGTTATAATTTGAAGTCACTACAGAGACATAAGCATTTTCATTCTGATAGAGATTCTGTTGTTCCTTACTGTTAGACGGGTGATGAATACCCCACAGTACAAGGACTTCTTTCCCTTTTTTGTTCACATAAGAATTTTTCAGCTTTGGGTATGAGCCCTCCTTCTCCGTCAGCCAAAGCAAATTTCTGTAAAAACTGCTTTTCCCCTCATGGGAGCATGCTGCCGTTACTCCGTTTGTGTTGTGGTTGGGCCATGAGCTTTCTTTGGGAAATATTTCGAATCTTTCGAATGATGACACTGAGCTCAATTGCTCCCTCAGCTCCTCATAGTCGATGAAATCTCCTGGATAACATATTCCATTCTCAGAGTCTGGTGTTTCTACAATGTAGGACCATGATCTCACTGGAAGCAGTGGGTCGCATTCTGGATTTCCCAAGAGCCATCCGGCGATGTTACATTTCCCCAATTGTAGTGGGGCTATTCCTTTTAATCTACATAGTTTTCCGTTGTGGCTGTCTTCGAGCAGGTTAACAGAGTGTGTCACTGTCACATTCTTCTCGAGTACTGTGTCAACAGTGTCGGTTGAATTGTTCGCATGGTAGCCTATACATATTGTGTCTGCATCTGCAGCTGCAAGGCACATTACAG

> Primary HA gene sequence

CATGCTATTCTGCACTGCAAGATCCATTAGAACACATCCAGAAACTGATTGCCCCCAGGGAGACCAAAAGCACCAGTGAACTGGCGACAGTTGAGTAGATCGCCAGAATCTGATAGATCCCCATTGATTCCAATTTCACTCCATCTACCTTTTCCCTGTTCAACTTTGACTCTTCTGAATATTTGGGATAATCATAAGTCCCATTTCTTACACTTTCCATGCATTCATTGTCACACTTGTGGTAGAACTCAAAACATCCATTTCCGATTTCTTTGGCATTATTCTTTAATTGGCTTTTTACTTTCTCATACAGATTCTTCACATTTGAGTCATGGAAATCCAGAGTCCTTTCATTTTCCAGTAGAACTAACAATTCTGCATTATATGTCCAAATGTCCAGAAATCCATCATCAACTTTTTTATTTAAATTTTCCATCCTTTTTTCTAATTTGTTGAATTCTTTACCCACAGCTGTGAATTGAATGTTCATTTTCTCGATAACAGTGTTCACCTTGTTTGTAATCCCGTTAATGGCATTTTGTGTGCTTTTTTGATCCGCTGCATAGCCTGATCCCTGTTCATTCTGATGATGATAACCATACCATCCATCTATCATTCCAGTCCATCCCCCTTCAATAAAACCGGCAATGGCTCCAAATATACCTCTGGATTGAATGGACGGAATGTTCCTTAGTCCTGTAACCATCCTCAATTTGGCACTCCTGACGTATTTTGGGCACTCTCCTATTGTGACTGGGTGTATATTCTGGTAAGGGAGACTGCTGTTTATAGCTCCCAGGGGTGTTTGACACTTCGTGTTACACTCATGCATTGATGCGTTTGAAGTGATGATGCCGGACCCAAAGCCCTCTACTCAGTGCGAAAGCATACATTGGTGCTATTAGATTTCCATTTGCCTCAAATATTATTGTGTCTCCGGGTTTTAGCAAGGTCCAGTAATAGTTCATCCTTCCCAGTTTGATCTCTTACTTTGGGTCTTTCTGCTATTTCCGGGGTAAATCTCCTGTTATAATTTGAAGTCACTACAGAGACATAAGCATTTTCATTCTGATAGAGATTCTGTTGTTCCTTACTGTTAGACGGGTGATGAATACCCCACAGTACAAGGACTTCTTTCCCTTTTTTGTTCACATAAGAATTTTTCAGCTTTGGGTATGAGCCCTCCTTCTCCGTCAGCCATAGCAAATTTCTGTAAAAACTGCTTTTCCCCTCATGGGAGCATGCTGCCGTTACTCCGTTTGTGTTGTGGTTGGGCCATGAGCTTTCTTTGGGAAATATTTCGAATCTTTCGAATGATGACACTGAGCTCAATTGCTCCCTCAGCTCCTCATAGTCGATGAAATCTCCTGGATAACATATTCCATTCTCAGAGTTTGGTGTTTCTACAATGTAGGACCATGATCTCACTGGAAGCAGTGGGTCGCATTCTGGATTTCCCAAGAGCCATCCGGCGATGTTACATTTCCCCAATTGTAGTGGGGCTATTCCTTTTAATCTACATAGTTTTCCGTTGTGGCTGTCTTCGAGCAGGTTAACAGAGTGTGTCACTGTCACATTCTTCTCGAGTACTGTGTCAACAGTGTCGGTTGAATTGTTCGCATGGTAGCCTATACATATTGTGTCTGCATCTGCAGCTGCAAGGCACATACAGTC

>A/Puerto_Rico/8/1934|V01088|4

AGCAAAAGCAGGGGAAAATAAAAACAACCAAAATGAAGGCAAACCTACTGGTCCTGTTATGTGCACTTGCAGCTGCAGATGCAGACACAATATGTATAGGCTACCATGCGAACAATTCAACCGACACTGTTGACACAGTGCTCGAGAAGAATGTGACAGTGACACACTCTGTTAACCTGCTCGAAGACAGCCACAACGGAAAACTATGTAGATTAAAAGGAATAGCCCCACTACAATTGGGGAAATGTAACATCGCCGGATGGCTCTTGGGAAACCCAGAATGCGACCCACTGCTTCCAGTGAGATCATGGTCCTACATTGTAGAAACACCAAACTCTGAGAATGGAATATGTTATCCAGGAGATTTCATCGACTATGAGGAGCTGAGGGAGCAATTGAGCTCAGTGTCATCATTCGAAAGATTCGAAATATTTCCCAAAGAAAGCTCATGGCCCAACCACAACACAACCAAAGGAGTAACGGCAGCATGCTCCCATGCGGGGAAAAGCAGTTTTTACAGAAATTTGCTATGGCTGACGGAGAAGGAGGGCTCATACCCAAAGCTGAAAAATTCTTATGTGAACAAGAAAGGGAAAGAAGTCCTTGTACTGTGGGGTATTCATCACCCGTCTAACAGTAAGGATCAACAGAATATCTATCAGAATGAAAATGCTTATGTCTCTGTAGTGACTTCAAATTATAACAGGAGATTTACCCCGGAAATAGCAGAAAGACCCAAAGTAAGAGATCAAGCTGGGAGGATGAACTATTACTGGACCTTGCTAAAACCCGGAGACACAATAATATTTGAGGCAAATGGAAATCTAATAGCACCAAGGTATGCTTTCGCACTGAGTAGAGGCTTTGGGTCCGGCATCATCACCTCAAACGCATCAATGCATGAGTGTAACACGAAGTGTCAAACACCCCTGGGAGCTATAAACAGCAGTCTCCCTTTCCAGAATATACACCCAGTCACAATAGGAGAGTGCCCAAAATACGTCAGGAGTGCCAAATTGAGGATGGTTACAGGACTAAGGAACATTCCGTCCATTCAATCCAGAGGTCTATTTGGAGCCATTGCCGGTTTTATTGAAGGGGGATGGACTGGAATGATAGATGGATGGTACGGTTATCATCATCAGAATGAACAGGGATCAGGCTATGCAGCGGATCAAAAAAGCACACAAAATGCCATTAACGGGATTACAAACAAGGTGAACTCTGTTATCGAGAAAATGAACATTCAATTCACAGCTGTGGGTAAAGAATTCAACAAATTAGAAAAAAGGATGGAAAATTTAAATAAAAAAGTTGATGATGGATTTCTGGACATTTGGACATATAATGCAGAATTGTTAGTTCTACTGGAAAATGAAAGGACTCTGGATTTCCATGACTCAAATGTGAAGAATCTGTATGAGAAAGTAAAAAGCCAATTAAAGAATAATGCCAAAGAAATCGGAAATGGATGTTTTGAGTTCTACCACAAGTGTGACAATGAATGCATGGAAAGTGTAAGAAATGGGACTTATGATTATCCCAAATATTCAGAAGAGTCAAAGTTGAACAGGGAAAAGGTAGATGGAGTGAAATTGGAATCAATGGGGATCTATCAGATTCTGGCGATCTACTCAACTGTCGCCAGTTCACTGGTGCTTTTGGTCTCCCTGGGGGCAATCAGTTTCTGGATGTGTTCTAATGGATCTTTGCAGTGCAGAATATGCATCTGAGATTAGAATTTCAGAAATATGAGGAAAAACACCCTTGTTTCTACT

>A/Puerto_Rico/8/1934|LC120391|4

AGCAAAAGCAGGGGAAAATAAAAACAACCAAAATGAAGGCAAACCTACTGGTCCTGTTATGTGCACTTGCAGCTGCAGATGCAGACACAATATGTATAGGCTACCATGCGAACAATTCAACCGACACTGTTGACACAGTACTCGAGAAGAATGTGACAGTGACACACTCTGTTAACCTGCTCGAAGACAGCCACAACGGAAAACTATGTAGATTAAAAGGAATAGCCCCACTACAATTGGGGAAATGTAACATCGCCGGATGGCTCTTGGGAAACCCAGAATGCGACCCACTGCTTCCAGTGAGATCATGGTCCTACATTGTAGAAACACCAAGCTCTGAGAATGGAATATGTTATCCAGGAGATTTCATCGACTATGAGGAGCTGAAGGAGCAATTGAGCTCAGTGTCATCATTCGAAAGATTCGAAATATTTCCCAAAGAAAGCTCATGGCCCAACCACAACACAAACAAAGGAGTAACGGCAGCATGCTCCCATGAGGGGAAAAGCAGTTTTTACAGAAATTTGCTATGGCTGACGGAGAAGGAGGGCTCATACCCAAAGCTGAAAAATTCTTATGTGAACAAGAAAGGGAAAGAAGTCCTTGTACTGTGGGGTATTCATCACCCGTCTAACAGTAAGGAACAACAGAATCTCTATCAGAATGAAAATGCTTATGTCTCTGTAGTGACTTCAAATTATAACAGGAGATTTACCCCGGAAATAGCAGAAAGACCCAAAGTAAAAGATCAAGCTGGGAGGATGAACTATTACTGGACCTTGCTAAAACCCGGAGACACAATAATATTTGAGGCAAATGGAAATCTAATAGCACCAATGTATGCTTTCGCACTGAGTAGAGGCTTTGGGTCCGGCATCATCACCTCAAACGCATCAATGCATGAGTGTAACACGAAGTGTCAAACACCCCTGGGAGCTATAAACAGCAGTCTCCCTTTCCAGAATATACACCCAGTCACAATAGGAGAGTGCCCAAAATACGTCAGGAGTGCCAAATTGAGGATGGTTACAGGACTAAGGAACATTCCGTCCATCCAATCCAGAGGTCTATTTGGAGCCATTGCCGGTTTTATTGAAGGGGGATGGACTGGAATGATAGATGGATGGTATGGTTATCATCATCAGAATGAACAGGGATCAGGCTATGCAGCGGATCAAAAAAGCACACAAAATGCCGTTAACGGGATTACAAACAAGGTGAACACTGTTATCGAGAAAATGAACATTCAATTCACAGCTGTGGGTAAAGAATTCAACAAATTAGAAAAGAGGATGGAAAATTTAAATAAAAAAGTTGATGATGGATTTCTGGACATTTGGACATATAATGCAGAATTGTTAGTTCTACTGGAAAATGAAAGGACTCTGGATTTCCATGACTCAAATGTGAAGAATCTGTATGAGAAAGTAAAAAGCCAATTAAAGAATAATGCCAAAGAAATCGGAAATGGATGTTTTGAGTTCTACCACAAGTGTGACAATGAATGCATGGAAAGTGTAAGAAATGGGACTTATGATTATCCCAAATATTCAGAAGAGTCAAAGTTGAACAGGGAAAAGGTAGATGGAGTGAAATTGGAATCAATGGGGATCTATCAGATTCTGGCGATCTACTCAACTGTCGCCAGTTCACTGGTGCTTTTGGTCTCCCTGGGGGCAATCAGTTTCTGGATGTGTTCTAATGGATCTTTGCAGTGCAGAATATGCATCTAGATTAGAATTTCAGAAATATGAGGAAAAACACCCTTGTTTCTACT

>A/Puerto_Rico/8/1934|MH785011|4

AGCAAAAGCAGGGGAAAATAAAAACAACCAAAATGAAGGCAAACCTACTGGTCCTGTTATGTGCACTTGCAGCTGCAGATGCAGACACAATATGTATAGGCTACCATGCGAACAATTCAACCGACACTGTTGACACAGTACTAGAGAAGAATGTGACAGTGACACACTCTGTTAACCTGCTCGAAGACAGCCACAACGGAAAACTATGTAGATTAAAAGGAATAACCCCACTACAATTGGGGAATTGTAACATCGCCGGATGGCTCTTGGGAAACCCAGAATGCGACCCACTGCTTCCAGTGAGATCATGGTCCTACATTGTAGAAACACCAAACTCTGAGAATGGAATATGTTATCCAGGAGATTTCATCGACTATGAAGAACTGAGGGAGCAATTGAGCTCAGTGTCATCATTCGAAAGATTCGAAATATTTCCCAAAGAAAGCTCATGGCCCAACCACAACACAAACAAAGGAGTAACGGCAGCATGCTCCCATGCGGGGAAAAGCAGTTTTTACAGAAATTTGCTATGGCTGACGGAGAAGGAGGGCTCATACCCAAAGCTGAAAAATTCTTATGTGAACAAGAAAGGGAAGGAAGTCCTTGTACTGTGGGGTATTCATCACCCGTCTAACAGTAAGGAACAACAGAATCTCTATCAGAATGAAAATGCTTATGTCTCTGTAGTGACTTCAAATTATAACAGGAGATTTACCCCGGAAATAGCAGAAAGACCCAAAGTAAGAGATCAAGCTGGGAGGATGAACTATTACTGGACCTTGCTAAAACCCGGAGACACAATAATATTTGAGGCAAATGGAAATCTAATAGCACCAAGGTATGCTTTCGCACTGAGTAGAGGCTTTGGGTCCGGCATCATCACCTCAAACGCATCAATGCATGAGTGTAACACGAAGTGTCAAACACCCCTGGGAGCTATAAACAGCAGTCTCCCTTTCCAGAATATACACCCAGTCACAATAGGAGAGTGCCCAAAATACGTCAGGAGTGCCAAATTGAGGATGGTTACAGGACTAAGGAACATTCCGTCCATTCAATCCAGAGGTCTATTTGGAGCCATTGCCGGTTTTATTGAAGGTGGATGGACTGGAATGATAGATGGATGGTATGGTTATCATCATCAGAATGAACAGGGATCAGGCTATGCAGCGGATCAAAAAAGCACACAAAATGCCATTAACGGGATTACAAACAAGGTGAACTCTGTTATCGAGAAAATGAACACTCAATTCACAGCTGTGGGTAAAGAATTCAACAAATTAGAAAAAAGGATGGAAAATTTAAATAAAAAAGTTGATGATGGATTTCTGGACATTTGGACATATAATGCAGAATTGTTAGTTCTACTGGAAAATGAAAGGACTCTGGATTTCCATGACTCAAATGTGAAGAATCTGTATGAGAAAGTAAAAAGCCAATTAAAGAATAATGCCAAAGAAATCGGAAATGGATGTTTTGAGTTCTACCACAAGTGTGACAATGAATGCATGGAAAGTGTAAGAAATGGGACTTATGATTATCCCAAATATTCAGAAGAGTCAAAGTTGAACAGGGAAAAGGTAGATGGAGTGAAATTGGAATCAATGGGGATCTATCAGATTCTGGCGATCTACTCAACTGTCGCCAGTTCACTGGTGCTTTTGGTCTCCCTGGGGGCAATCAGTTTCTGGATGTGTTCTAATGGATCTTTGCAGTGCAGAATATGCATCTGAGATTAGAATTTCAGAAATATGAGGAAAAACACCCTTGTTTCTACT

>A/Puerto_Rico/8/1934|CY148243|4

AAAATGAAGGCAAACCTACTGGTCCTGTTAAGTGCACTTGCAGCTGCAGATGCAGACACAATATGTATAGGCTACCATGCGAACAATTCAACCGACACTGTTGACACAGTACTCGAGAAGAATGTGACAGTGACACACTCTGTTAACCTGCTCGAAGACAGCCACAACGGAAAACTATGTAGATTAAAAGGAATAGCCCCACTACAATTGGGGAAATGTAACATCGCCGGATGGCTCTTGGGAAACCCAGAATGCGACCCACTGCTTCCAGTGAGATCATGGTCCTACATTGTAGAAACACCAAACTCTGAGAATGGAATATGTTATCCAGGAGATTTCATCGACTATGAGGAGCTGAGGGAGCAATTGAGCTCAGTGTCATCATTCGAAAGATTCGAAATATTTCCCAAAGAAAGCTCATGGCCCAACCACAACACAAACGGAGTAACGGCAGCATGCTCCCATGAGGGGAAAAGCAGTTTTTACAGAAATTTGCTATGGCTGACGGAGAAGGAGGGCTCATACCCAAAGCTGAAAAATTCTTATGTGAACAAAAAAGGGAAAGAAGTCCTTGTACTGTGGGGTATTCATCACCCGCCTAACAGTAAGGAACAACAGAATCTCTATCAGAATGAAAATGCTTATGTCTCTGTAGTGACTTCAAATTATAACAGGAGATTTACCCCGGAAATAGCAGAAAGACCCAAAGTAAGAGATCAAGCTGGGAGGATGAACTATTACTGGACCTTGCTAAAACCCGGAGACACAATAATATTTGAGGCAAATGGAAATCTAATAGCACCAATGTATGCTTTCGCACTGAGTAGAGGCTTTGGGTCCGGCATCATCACCTCAAACGCATCAATGCATGAGTGTAACACGAAGTGTCAAACACCCCTGGGAGCTATAAACAGCAGTCTCCCTTACCAGAATATACACCCAGTCACAATAGGAGAGTGCCCAAAATACGTCAGGAGTGCTAAATTGAGGATGGTTACAGGACTAAGGAACACTCCGTCCATTCAATCCAGAGGTCTATTTGGAGCCATTGCCGGTTTTATTGAAGGGGGATGGACTGGAATGATAGATGGATGGTATGGTTATCATCATCAGAATGAACAGGGATCAGGCTATGCAGCGGATCAAAAAAGCACACAAAATGCCATTAACGGGATTACAAACAAGGTGAACACTGTTATCGAGAAAATGAACATTCAATTCACAGCTGTGGGTAAAGAATTCAACAAATTAGAAAAAAGGATGGAAAATTTAAATAAAAAAGTTGATGATGGATTTCTGGACATTTGGACATATAATGCAGAATTGTTAGTTCTACTGGAAAATGAAAGGACTCTGGATTTCCATGACTCAAATGTGAAGAATCTGTATGAGAAAGTAAAAAGCCAATTAAAGAATAATGCCAAAGAAATCGGAAATGGATGTTTTGAGTTCTACCACAAGTGTGACAATGAATGCATGGAAAGTGTAAGAAATGGGACTTATGATTATCCCAAATATTCAGAAGAGTCAAAGTTGAACAGGGAAAAGGTAGATGGAGTGAAATTGGAATCAATGGGGATCTATCAGATTCTGGCGATCTACTCAACTGTCGCCAGTTCACTGGTGCTTTTGGTCTCCCTGGGGGCAATCAGTTTCTGGATGTGTTCTAATGGATCTTTGCAGTGCAGAATATGCATCTGAGATTAGAATTTCAGAAATATGAG

>A/Puerto_Rico/8/1934|HE802058|4

AGCAAAAGCAGGGGAAAATAAAAACAACCAAAATGAAGGCAAACCTACTGGTCCTGTTATGTGCACTTGCAGCTGCAGATGCAGACACAATATGTATAGGCTACCATGCGAACAATTCAACCGACACTGTTGACACAGTACTCGAGAAGAATGTGACAGTGACACACTCTGTTAACCTGCTCGAAGACAGCCACAACGGAAAACTATGTAGATTAAAAGGAATAGCCCCACTACAATTGGGGAAATGTAACATCGCCGGATGGCTCTTGGGAAACCCAGAATGCGACCCACTGCTTCCAGTGAGATCATGGTCCTACATTGTAGAAACACCAAACTCTGAGAATGGAATATGTTATCCAGGAGATTTCATCGACTATGAGGAGCTGAGGGAGCAATTGAGCTCAGTGTCATCATTCGAAAGATTCGAAATATTTCCCAAAGAAAGCTCATGGCCCAACCACAACACAAACGGAGTAACGGCAGCATGCTCCCATGAGGGGAAAAGCAGTTTTTACAGAAATTTGCTATGGCTGACGGAGAAGGAGGGCTCATACCCAAAGCTGAAAAATTCTTATGTGAACAAAAAAGGGAAAGAAGTCCTTGTACTGTGGGGTATTCATCACCCGCCTAACAGTAAGGAACAACAGAATCTCTATCAGAATGAAAATGCTTATGTCTCTGTAGTGACTTCAAATTATAACAGGAGATTTACCCCGGAAATAGCAGAAAGACCCAAAGTAAGAGATCAAGCTGGGAGGATGAACTATTACTGGACCTTGCTAAAACCCGGAGACACAATAATATTTGAGGCAAATGGAAATCTAATAGCACCAATGTATGCTTTCGCACTGAGTAGAGGCTTTGGGTCCGGCATCATCACCTCAAACGCATCAATGCATGAGTGTAACACGAAGTGTCAAACACCCCTGGGAGCTATAAACAGCAGTCTCCCTTACCAGAATATACACCCAGTCACAATAGGAGAGTGCCCAAAATACGTCAGGAGTGCCAAATTGAGGATGGTTACAGGACTAAGGAACATTCCGTCCATTCAATCCAGAGGTCTATTTGGAGCCATTGCCGGTTTTATTGAAGGGGGATGGACTGGAATGATAGATGGATGGTATGGTTATCATCATCAGAATGAACAGGGATCAGGCTATGCAGCGGATCAAAAAAGCACACAAAATGCCATTAACGGGATTACAAACAAGGTGAACACTGTTATCGAGAAAATGAACATTCAATTCACAGCTGTGGGTAAAGAATTCAACAAATTAGAAAAAAGGATGGAAAATTTAAATAAAAAAGTTGATGATGGATTTCTGGACATTTGGACATATAATGCAGAATTGTTAGTTCTACTGGAAAATGAAAGGACTCTGGATTTCCATGACTCAAATGTGAAGAATCTGTATGAGAAAGTAAAAAGCCAATTAAAGAATAATGCCAAAGAAATCGGAAATGGATGTTTTGAGTTCTACCACAAGTGTGACAATGAATGCATGGAAAGTGTAAGAAATGGGACTTATGATTATCCCAAATATTCAGAAGAGTCAAAGTTGAACAGGGAAAAGGTAGATGGAGTGAAATTGGAATCAATGGGGATCTATCAGATTCTGGCGATCTACTCAACTGTCGCCAGTTCACTGGTGCTTTTGGTCTCCCTGGGGGCAATCAGTTTCTGGATGTGTTCTAATGGATCTTTGCAGTGCAGAATATGCATCTGAGATTAGAATTTCAGAGATATGAGGAAAACACCCTTGTTTCTACT

>A/Puerto_Rico/8/1934|CY121109|4

GGAAAATAAAAACAACCAAAATGAAGGCAAACCTACTGGTCCTGTTATGTGCACTTGCAGCTGCAGATGCAGACACAATATGTATAGGCTACCATGCGAACAATTCAACCGACACTGTTGACACAGTACTCGAGAAGAATGTGACAGTGACACACTCTGTTAACCTGCTCGAAGACAGCCACAACGGAAAACTATGTAGATTAAAAGGAATAGCCCCACTACAATTGGGGAAATGTAACATCGCCGGATGGCTCTTGGGAAACCCAGAATGCGACCCACTGCTTCCAGTGAGATCATGGTCCTACATTGTAGAAACACCAAACTCTGAGAATGGAATATGTTATCCAGGAGATTTCATCGACTATGAGGAGCTGAGGGAGCAATTGAGCTCAGTGTCATCATTCGAAAGATTCGAAATATTTCCCAAAGAAAGCTCATGGCCCAACCACAACACAAACGGAGTAACGGCAGCATGCTCCCATGAGGGGAAAAGCAGTTTTTACAGAAATTTGCTATGGCTGACGGAGAAGGAGGGCTCATACCCAAAGCTGAAAAATTCTTATGTGAACAAAAAAGGGAAAGAAGTCCTTGTACTGTGGGGTATTCATCACCCGTCTAACAGTAAGGAACAACAGAATCTCTATCAGAATGAAAATGCTTATGTCTCTGTAGTGACTTCAAATTATAACAGGAGATTTACCCCGGAAATAGCAGAAAGACCCAAAGTAAGAGATCAAGCTGGGAGGATGAACTATTACTGGACCTTGCTAAAACCCGGAGACACAATAATATTTGAGGCAAATGGAAATCTAATAGCACCAATGTATGCTTTCGCACTGAGTAGAGGCTTTGGGTCCGGCATCATCACCTCAAACGCATCAATGCATGAGTGTAACACGAAGTGTCAAACACCCCTGGGAGCTATAAACAGCAGTCTCCCTTACCAGAATATACACCCAGTCACAATAGGAGAGTGCCCAAAATACGTCAGGAGTGCCAAATTGAGGATGGTTACAGGACTAAGGAACATTCCGTCCATTCAATCCAGAGGTCTATTTGGAGCCATTGCCGGTTTTATTGAAGGGGGATGGACTGGAATGATAGATGGATGGTATGGTTATCATCATCAGAATGAACAGGGATCAGGCTATGCAGCGGATCAAAAAAGCACACAAAATGCCATTAACGGGATTACAAACAAGGTGAACACTGTTATCGAGAAAATGAACATTCAATTCACAGCTGTGGGTAAAGAATTCAACAAATTAGAAAAAAGGATGGAAAATTTAAATAAAAAAGTTGATGATGGATTTCTGGACATTTGGACATATAATGCAGAATTGTTAGTTCTACTGGAAAATGAAAGGACTCTGGATTTCCATGACTCAAATGTGAAGAATCTGTATGAGAAAGTAAAAAGCCAATTAAAGAATAATGCCAAAGAAATCGGAAATGGATGTTTTGAGTTCTACCACAAGTGTGACAATGAATGCATGGAAAGTGTAAGAAATGGGACTTATGATTATCCCAAATATTCAGAAGAGTCAAAGTTGAACAGGGAAAAGGTAGATGGAGTGAAATTGGAATCAATGGGGATCTATCAGATTCTGGCGATCTACTCAACTGTCGCCAGTTCACTGGTGCTTTTGGTCTCCCTGGGGGCAATCAGTTTCTGGATGTGTTCTAATGGATCTTTGCAGTGCAGAATATGCATCTGAGATTAGAATTTCAGAGATATGAGGAAAAACAC

>A/Puerto_Rico/8/1934|CY009444|4

GAAAATAAAAACAACCAAAATGAAGGCAAACCTACTGGTCCTGTTATGTGCACTTGCAGCTGCAGATGCAGACACAATATGTATAGGCTACCATGCGAACAATTCAACCGACACTGTTGACACAGTACTCGAGAAGAATGTGACAGTGACACACTCTGTTAACCTGCTCGAAGACAGCCACAACGGAAAACTATGTAGATTAAAAGGAATAGCCCCACTACAATTGGGGAAATGTAACATCGCCGGATGGCTCTTGGGAAACCCAGAATGCGACCCACTGCTTCCAGTGAGATCATGGTCCTACATTGTAGAAACACCAAACTCTGAGAATGGAATATGTTATCCAGGAGATTTCATCGACTATGAGGAGCTGAGGGAGCAATTGAGCTCAGTGTCATCATTCGAAAGATTCGAAATATTTCCCAAAGAAAGCTCATGGCCCAACCACAACACAAACGGAGTAACGGCAGCATGCTCCCATGAGGGGAAAAGCAGTTTTTACAGAAATTTGCTATGGCTGACGGAGAAGGAGGGCTCATACCCAAAGCTGAAAAATTCTTATGTGAACAAAAAAGGGAAAGAAGTCCTTGTACTGTGGGGTATTCATCACCCGCCTAACAGTAAGGAACAACAGAATCTCTATCAGAATGAAAATGCTTATGTCTCTGTAGTGACTTCAAATTATAACAGGAGATTTACCCCGGAAATAGCAGAAAGACCCAAAGTAAGAGATCAAGCTGGGAGGATGAACTATTACTGGACCTTGCTAAAACCCGGAGACACAATAATATTTGAGGCAAATGGAAATCTAATAGCACCAATGTATGCTTTCGCACTGAGTAGAGGCTTTGGGTCCGGCATCATCACCTCAAACGCATCAATGCATGAGTGTAACACGAAGTGTCAAACACCCCTGGGAGCTATAAACAGCAGTCTCCCTTACCAGAATATACACCCAGTCACAATAGGAGAGTGCCCAAAATACGTCAGGAGTGCCAAATTGAGGATGGTTACAGGACTAAGGAACATTCCGTCCATTCAATCCAGAGGTCTATTTGGAGCCATTGCCGGTTTTATTGAAGGGGGATGGACTGGAATGATAGATGGATGGTATGGTTATCATCATCAGAATGAACAGGGATCAGGCTATGCAGCGGATCAAAAAAGCACACAAAATGCCATTAACGGGATTACAAACAAGGTGAACACTGTTATCGAGAAAATGAACATTCAATTCACAGCTGTGGGTAAAGAATTCAACAAATTAGAAAAAAGGATGGAAAATTTAAATAAAAAAGTTGATGATGGATTTCTGGACATTTGGACATATAATGCAGAATTGTTAGTTCTACTGGAAAATGAAAGGACTCTGGATTTCCATGACTCAAATGTGAAGAATCTGTATGAGAAAGTAAAAAGCCAATTAAAGAATAATGCCAAAGAAATCGGAAATGGATGTTTTGAGTTCTACCACAAGTGTGACAATGAATGCATGGAAAGTGTAAGAAATGGGACTTATGATTATCCCAAATATTCAGAAGAGTCAAAGTTGAACAGGGAAAAGGTAGATGGAGTGAAATTGGAATCAATGGGGATCTATCAGATTCTGGCGATCTACTCAACTGTCGCCAGTTCACTGGTGCTTTTGGTCTCCCTGGGGGCAATCAGTTTCTGGATGTGTTCTAATGGATCTTTGCAGTGCAGAATATGCATCTGAGATTAGAATTTCAGAGATATGAGGAAAAA

>A/Puerto_Rico/8/1934|AB671289|4

AGCAAAAGCAGGGGAAAATAAAAACAACCAAAATGAAGGCAAACCTACTGGTCCTGTTATGTGCACTTGCAGCTGCAGATGCAGACACAATATGTATAGGCTACCATGCGAACAATTCAACCGACACTGTTGACACAGTACTCGAGAAGAATGTGACAGTGACACACTCTGTTAACCTGCTCGAAGACAGCCACAACGGAAAACTATGTAGATTAAAAGGAATAGCCCCACTACAATTGGGGAAATGTAACATCGCCGGATGGCTCTTGGGAAACCCAGAATGCGACCCACTGCTTCCAGTGAGATCATGGTCCTACATTGTAGAAACACCAAACTCTGAGAATGGAATATGTTATCCAGGAGATTTCATCGACTATGAGGAGCTGAGGGAGCAATTGAGCTCAGTGTCATCATTCGAAAGATTCGAAATATTTCCCAAAGAAAGCTCATGGCCCAACCACAACACAAACGGAGTAACGGCAGCATGCTCCCATGAGGGGAAAAGCAGTTTTTACAGAAATTTGCTATGGCTGACGGAGAAGGAGGGCTCATACCCAAAGCTGAAAAATTCTTATGTGAACAAAAAAGGGAAAGAAGTCCTTGTACTGTGGGGTATTCATCACCCGCCTAACAGTAAGGAACAACAGAATCTCTATCAGAATGAAAATGCTTATGTCTCTGTAGTGACTTCAAATTATAACAGGAGATTTACCCCGGAAATAGCAGAAAGACCCAAAGTAAGAGATCAAGCTGGGAGGATGAACTATTACTGGACCTTGCTAAAACCCGGAGACACAATAATATTTGAGGCAAATGGAAATCTAATAGCACCAATGTATGCTTTCGCACTGAGTAGAGGCTTTGGGTCCGGCATCATCACCTCAAACGCATCAATGCATGAGTGTAACACGAAGTGTCAAACACCCCTGGGAGCTATAAACAGCAGTCTCCCTTACCAGAATATACACCCAGTCACAATAGGAGAGTGCCCAAAATACGTCAGGAGTGCCAAATTGAGGATGGTTACAGGACTAAGGAACATTCCGTCCATTCAATCCAGAGGTCTATTTGGAGCCATTGCCGGTTTTATTGAAGGGGGATGGACTGGAATGATAGATGGATGGTATGGTTATCATCATCAGAATGAACAGGGATCAGGCTATGCAGCGGATCAAAAAAGCACACAAAATGCCATTAACGGGATTACAAACAAGGTGAACACTGTTATCGAGAAAATGAACATTCAATTCACAGCTGTGGGTAAAGAATTCAACAAATTAGAAAAAAGGATGGAAAATTTAAATAAAAAAGTTGATGATGGATTTCTGGACATTTGGACATATAATGCAGAATTGTTAGTTCTACTGGAAAATGAAAGGACTCTGGATTTCCATGACTCAAATGTGAAGAATCTGTATGAGAAAGTAAAAAGCCAATTAAAGAATAATGCCAAAGAAATCGGAAATGGATGTTTTGAGTTCTACCACAAGTGTGACAATGAATGCATGGAAAGTGTAAGAAATGGGACTTATGATTATCCCAAATATTCAGAAGAGTCAAAGTTGAACAGGGAAAAGGTAGATGGAGTGAAATTGGAATCAATGGGGATCTATCAGATTCTGGCGATCTACTCAACTGTCGCCAGTTCACTGGTGCTTTTGGTCTCCCTGGGGGCAATCAGTTTCTGGATGTGTTCTAATGGATCTTTGCAGTGCAGAATATGCATCTGAGATTAGAATTTCAGAGATATGAGGAAAAACACCCTTGTTTCTACT

>A/Puerto_Rico/8/1934|CY105896|4

GAAAATAAAAACAACCAAAATGAAGGCAAACCTACTGGTCCTGTTATGTGCACTTGCAGCTGCAGATGCAGACACAATATGTATAGGCTACCATGCGAACAATTCAACCGACACTGTTGACACAGTACTCGAGAAGAATGTGACAGTGACACACTCTGTTAACCTGCTCGAAGACAGCCACAACGGAAAACTATGTAGATTAAAAGGAATAGCCCCACTACAATTGGGGAAATGTAACATCGCCGGATGGCTCTTGGGAAACCCAGAATGCGACCCACTGCTTCCAGTGAGATCATGGTCCTACATTGTAGAAACACCAAACTCTGAGAATGGAATATGTTATCCAGGAGATTTCATCGACTATGAGGAGCTGAGGGAGCAATTGAGCTCAGTGTCATCATTCGAAAGATTCGAAATATTTCCCAAAGAAAGCTCATGGCCCAACCACAACACAAACGGAGTAACGGCAGCATGCTCCCATGAGGGGAAAAGCAGTTTTTACAGAAATTTGCTATGGCTGACGGAGAAGGAGGGCTCATACCCAAAGCTGAAAAATTCTTATGTGAACAAAAAAGGGAAAGAAGTCCTTGTACTGTGGGGTATTCATCACCCGCCTAACAGTAAGGAACAACAGAATCTCTATCAGAATGAAAATGCTTATGTCTCTGTAGTGACTTCAAATTATAACAGGAGATTTACCCCGGAAATAGCAGAAAGACCCAAAGTAAGAGATCAAGCTGGGAGGATGAACTATTACTGGACCTTGCTAAAACCCGGAGACACAATAATATTTGAGGCAAATGGAAATCTAATAGCACCAATGTATGCTTTCGCACTGAGTAGAGGCTTTGGGTCCGGCATCATCACCTCAAACGCATCAATGCATGAGTGTAACACGAAGTGTCAAACACCCCTGGGAGCTATAAACAGCAGTCTCCCTTACCAGAATATACACCCAGTCACAATAGGAGAGTGCCCAAAATACGTCAGGAGTGCCAAATTGAGGATGGTTACAGGACTAAGGAACATTCCGTCCATTCAATCCAGAGGTCTATTTGGAGCCATTGCCGGTTTTATTGAAGGGGGATGGACTGGAATGATAGATGGATGGTATGGTTATCATCATCAGAATGAACAGGGATCAGGCTATGCAGCGGATCAAAAAAGCACACAAAATGCCATTAACGGGATTACAAACAAGGTGAACACTGTTATCGAGAAAATGAACATTCAATTCACAGCTGTGGGTAAAGAATTCAACAAATTAGAAAAAAGGATGGAAAATTTAAATAAAAAAGTTGATGATGGATTTCTGGACATTTGGACATATAATGCAGAATTGTTAGTTCTACTGGAAAATGAAAGGACTCTGGATTTCCATGACTCAAATGTGAAGAATCTGTATGAGAAAGTAAAAAGCCAATTAAAGAATAATGCCAAAGAAATCGGAAATGGATGTTTTGAGTTCTACCACAAGTGTGACAATGAATGCATGGAAAGTGTAAGAAATGGGACTTATGATTATCCCAAATATTCAGAAGAGTCAAAGTTGAACAGGGAAAAGGTAGATGGAGTGAAATTGGAATCAATGGGGATCTATCAGATTCTGGCGATCTACTCAACTGTCGCCAGTTCACTGGTGCTTTTGGTCTCCCTGGGGGCAATCAGTTTCTGGATGTGTTCTAATGGATCTTTGCAGTGCAGAATATGCATCTGAGATTAGAATTTCAGAGATATGAGGA
